# Virtual Screening Approaches to Identify Promising Multitarget-Directed Ligands for the Treatment of Autism Spectrum Disorder

**DOI:** 10.3390/molecules29225271

**Published:** 2024-11-07

**Authors:** Jakub Jończyk, Klaudia Przybylska, Marek Staszewski, Justyna Godyń, Tobias Werner, Monika Stefaniak-Napieralska, Holger Stark, Krzysztof Walczyński, Marek Bajda

**Affiliations:** 1 Department of Physicochemical Drug Analysis, Faculty of Pharmacy, Jagiellonian University Medical College, 30-688 Kraków, Poland; jakub.jonczyk@uj.edu.pl (J.J.); justyna.godyn@uj.edu.pl (J.G.); 2 Sano—Centre for Computational Medicine, 30-054 Kraków, Poland; 3 Department of Synthesis and Technology of Drugs, Faculty of Pharmacy, Medical University of Lodz, 90-151 Lodz, Poland; marek.staszewski@umed.lodz.pl (M.S.); monika.stefaniak@umed.lodz.pl (M.S.-N.); krzysztof.walczynski@umed.lodz.pl (K.W.); 4 Institute of Pharmaceutical and Medicinal Chemistry, Heinrich Heine University, 40225 Düsseldorf, Germany; t.werner@hhu.de (T.W.); stark@hhu.de (H.S.)

**Keywords:** autism spectrum disorder, virtual screening, histamine H_3_ receptor, multitarget-directed ligands, dopamine receptors, cholinesterases

## Abstract

Autism spectrum disorder is a complex neurodevelopmental disorder. The available medical treatment options for autism spectrum disorder are very limited. While the etiology and pathophysiology of autism spectrum disorder are still not fully understood, recent studies have suggested that wide alterations in the GABAergic, glutamatergic, cholinergic, and serotonergic systems play a key role in its development and progression. Histamine neurotransmission is known to have complex interactions with other neurotransmitters that fit perfectly into the complex etiology of this disease. Multitarget-directed compounds with an affinity for the histamine H_3_ receptor indicate an interesting profile of activity against autism spectrum disorder in animal models. Here, we present the results of our research on the properties of (4-piperazin-1-ylbutyl)guanidine derivatives acting on histamine H_3_ receptors as potential multitarget ligands. Through the virtual screening approach, we identified promising ligands among 32 non-imidazole histamine H_3_ receptor antagonists/inverse agonists with potential additional activity against the dopamine D_2_ receptor and/or cholinesterases. The virtual screening protocol integrated predictions from SwissTargetPrediction, SEA, and PPB2 tools, along with molecular docking simulations conducted using GOLD 5.3 and Glide 7.5 software. Among the selected ligands, compounds **25** and **30** blocked radioligand binding to the D_2_ receptor at over 50% at a screening concentration of 1 µM. Further experiments allowed us to determine the p*K*_i_ value at the D_2_ receptor of 6.22 and 6.12 for compounds **25** and **30**, respectively. Our findings suggest that some of the tested compounds could be promising multitarget-directed ligands for the further research and development of more effective treatments for autism spectrum disorder.

## 1. Introduction

Autism spectrum disorder (ASD) is a multifaceted neurodevelopmental disorder that affects communication, social interaction, and behavior [1]. According to the Centers for Disease Control (CDC) in 2020, 1 in 36 children has been identified with ASD in the United States alone [2]. The global prevalence of ASD is progressively rising year after year [3]. ASD can significantly affect a person’s life, leading to social and communication difficulties. The available treatment options for ASD appear to be very limited. While some behavioral therapies have shown promise in improving the outcomes for individuals with ASD, there are still unmet needs, particularly for severe forms of ASD, which require more intensive interventions [4]. Therapy mainly focuses on managing the associated symptoms, so there is a need to develop remedies that target the underlying symptomatic neurobiological and behavioral mechanisms of ASD [4,5].

ASD is a heterogeneous disorder with a complex etiology involving genetic and environmental factors leading to structural and functional abnormalities in the brain, alterations in neurotransmitter systems, and immune dysfunctions [5,6,7]. However, actual research sheds some light on the pathophysiology of ASD. The most studied neurotransmitter systems in ASD are the GABAergic and glutamatergic systems, which are involved in the regulation of neuronal excitability. Excitatory/inhibitory imbalance in ASD was observed in key brain regions such as the neocortex, hippocampus, amygdala, and cerebellum [8,9,10,11]. This mainly leads to a reduction in the levels of GABA released and amount of GABA receptors with co-occurring increased levels of glutamate and reduced activity of glutamate transporters.

The complexity of the factors contributing to ASD mirrors the complexity in the interactions between different neurotransmitter systems [12]. In the case of the GABA and glutamatergic systems [8,13,14], there are many premises linking their neurotransmission with the histamine, dopamine, or cholinergic systems [15,16,17,18,19].

The brain histaminergic system controls many essential physiological functions, and its dysfunction is related to several neuropsychiatric disorders [20,21,22]. Histamine works by binding to four histamine receptor subtypes, including the H_3_ receptors, which regulate histamine synthesis and release by a negative feedback mechanism. These G_i/o_-coupled inhibitory receptors, predominantly expressed in the brain, also control the release of other neurotransmitters in the CNS [23]. Therefore, histamine H_3_ receptor (H_3_R) antagonists are considered for use in treating various brain disorders, including Alzheimer’s disease, schizophrenia, and narcolepsy. It was observed that prenatal exposure to valproic acid (one of the anticonvulsants) in zebrafish was associated with development of autism spectrum disorder (ASD)-like symptoms, involving impaired sociability and stereotypies. These studies also revealed decreased levels of H_3_R and histidine decarboxylase and a decreased number of histaminergic neurons [24]. H_3_R antagonists have been found to improve behavioral deficiencies in animal models of schizophrenia and ASD [20,25,26]. Famotidine, a histamine H_2_ receptor antagonist, has been suggested as a possible treatment for children with ASD because it alleviates sociability deficits [27,28]. Ciproxifan, a first-generation H_3_R antagonist, has been shown to attenuate impaired sociability and stereotypies in an animal model of ASD [29]. Similar results were obtained by subchronic treatment with the potent and selective H_3_R antagonist DL77 in a prenatal valproic acid-induced mouse model of autism [30].

The dopamine system is associated with reward processing and its disruption has been implicated in neuropsychiatric disorders, including ADHD and ASD [31,32,33]. Recent studies have reported reduced dopaminergic signaling in ASD patients, highlighting reward-processing deficits for both social and nonsocial rewards [34,35]. Another clinical study showed reduced dopamine levels in the medial prefrontal cortex of medication-free ASD patients, which suggests an aberrant function of the dopaminergic systems in ASD [36]. In a BTBR (Black and Tan Brachyury) mice model, significant reductions in both pre- and postsynaptic dopamine D_2_ receptors (D_2_R) and adenosine A_2A_ receptor functions were observed [37]. Similar to H_3_R, D_2_R is a G_i/o_-coupled inhibitory receptor and, as an autoreceptor, it regulates the levels of dopamine in the synaptic cleft [38]. Research has demonstrated that the utilization of D_2_ antagonists enhances dopamine availability in the prefrontal cortex and striatum of BTBR mice, thereby contributing to the mitigation of autistic behavior [39]. One study revealed that specific single-nucleotide polymorphisms (SNPs) within the dopamine receptor D_2_ gene are significantly correlated with a heightened risk of ASD in children [40]. Another study found an increase in D_3_ receptor mRNA levels in the basal ganglia of individuals with ASD. This disruption is believed to contribute to the motor dysfunctions and stereotypies observed in ASD [41].

Although atypical neuroleptics, risperidone and aripiprazole, are the only drugs with consistent clinical efficacy in ASD, their effects on the core symptoms of ASD are still being studied [42,43]. The use of typical neuroleptics in the treatment of autism spectrum disorder is limited to managing severe behavioral problems with haloperidol [44]. Since autistic-like behavior arises from dopaminergic dysfunction, the study of dopaminergic dysfunction is vital for the neurodevelopmental disorder. Studies have indicated the presence of an H_3_R-D_2_R complex in the spiny projection neurons of the striatum. Notably, the activation of H_3_Rs using specific agonists has been shown to effectively counteract locomotor activity induced by D_2_R agonists, thus highlighting a direct interaction between these receptors [45].

In addition to histaminergic and dopaminergic systems, dysfunction in the cholinergic system is observed in both humans and animal models of ASD [16]. There are abnormalities in the number and structure of neurons in a basal forebrain cholinergic nucleus of ASD patients, as well as reduced levels of choline and muscarinic receptors in several brain regions. Therefore, it can be assumed that the cholinergic system plays a role in controlling ASD-related behaviors, such as attention, cognitive flexibility, social interaction, and stereotypical behaviors. Acetyl- and butyrylcholinesterases play a key role in signal termination within this system. Inhibiting AChE has emerged as a potential therapeutic strategy for managing cognitive-related symptoms [46]. Clinical studies exploring AChE inhibitors like donepezil have shown improvements in specific behaviors, such as reduced irritability or enhanced communication [47]. Similar to the interaction between H_3_R and D_2_Rs, the inhibition of acetylcholine release has been observed in cholinergic neurons following H_3_R activation [48].

**Figure 1:**
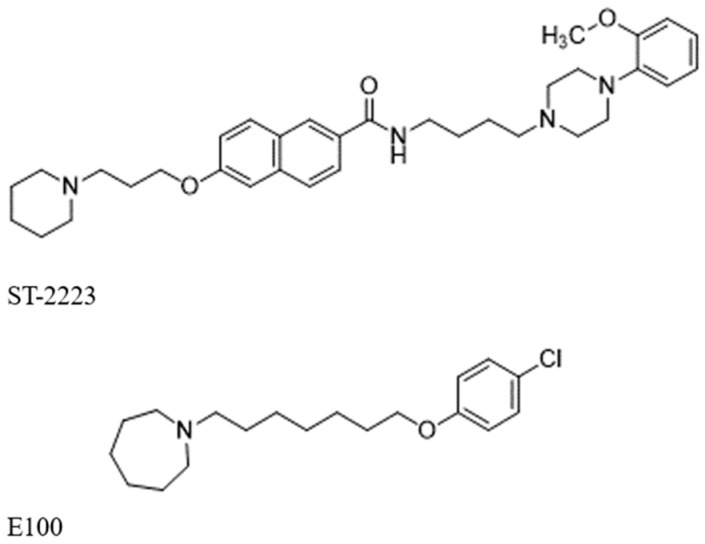
Structures of **ST-2223**, acting as histamine H_3_, D_2_/D_3_ receptor antagonists, and **E100**, H_3_R antagonist with acetylcholinesterase inhibitory activity.

Due to such a multifaceted pathomechanism, the potential therapy for ASD seems to be an ideal opportunity for the use of multitarget-directed ligands. Current approaches for neuropsychiatric disorders include both the polypharmacology of targeted receptors as well as the designing of one multitarget compound with activity against more than one biological target [49,50]. The first multitarget-directed ligands (MTDLs) combining activity on H_3_ receptors with affinity at the dopamine D_2_ and D_3_ receptors have already been identified [39]. Test results for **ST-2223** in mouse ASD models showed significant improvement in repetitive and compulsive behaviors by reducing the increased percentage of marbles buried in marble-burying behavior (MBB) [39]. Similar results were demonstrated by the compound **E100**, which is a simultaneous acetylcholinesterase (AChE) inhibitor and H_3_R antagonist [48,51]. The structures of these multitarget compounds are shown in Figure 1.

**Figure 2:**
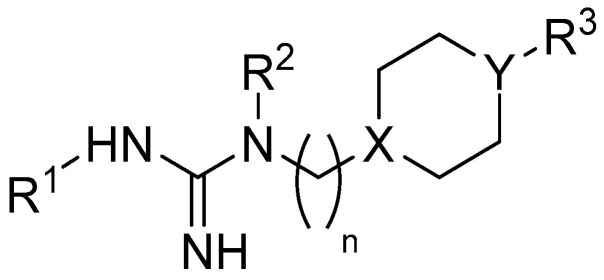
The common structural pattern in the majority of the compounds that underwent testing. X and Y are either CH or N atoms; *n* ranges from 2 to 6 carbon atoms.

Inspired by these findings, we attempted to uncover MTDLs with potential application in ASD in a group of 32 non-imidazole guanidine-based H_3_R antagonists/inverse agonists [52,53]. This series of *N*-substituted-*N*-[ω-(ω-phenoxyalkylpiperazin-1-yl)alkyl]guanidine derivatives was inspired by the structures of known histamine receptor ligands: impromidine and JB 98064 [54,55]. While the activity against H_3_R for the compounds has been established in previous studies, their capability to function as MTDL compounds has yet to be determined. The general structure of the tested compounds is shown in Figure 2. The detailed structures of the compounds are summarized in Table S1, which is included in the Supplementary Materials.

Taking into account earlier findings regarding the use of H_3_R-based MTDLs to mitigate autistic-like behaviors in mice, our primary emphasis was on the exploration of compounds with potential affinity for D_2_R. We also acknowledged the potential of inhibiting acetyl- and butyrylcholinesterase (BChE) with simultaneous H_3_R antagonism in regulating ASD-related behaviors, which led us to examine the inhibitory activity of selected compounds against these enzymes. Our choice was to utilize a virtual screening protocol to identify compounds that display the expected activity spectrum. The adapted protocol combined tools that consider the ligand’s structure to predict its potential biological targets with molecular docking, a technique that assesses the binding of ligands to the biological targets based on their structure. Following the virtual screening results, we chose the most promising compounds. Their activities against D_2_R and cholinesterases were assessed through suitable in vitro experiments.

## 2. Results and Discussion

We started with a computer-aided analysis of the compounds, assessing their potential activity. This analysis was based on three known biological target prediction models: SwissTargetPrediction [56], SEA [57], and PPB2 [58]. Each of them uses a different algorithm that assigns probable biological activity to compounds based on their structure. Both SEA and PPB2 predictors identified H_3_R, D_2_R, and cholinesterase activities among the 15 most plausible biological targets for the analyzed compounds. Interestingly, in many cases, affinity at the D_2_R was indicated to be more likely than that at the H_3_R. On the other hand, both predictors indicated a low probability of affinity of the tested ligands towards AChE or BChE. SwissTargetPredictor turned out to be much more restrictive. Only 11 of the tested compounds were indicated as potentially affine at the H_3_ histamine receptor, 7 compounds as affine at the D_2_R, and 3 against cholinesterases. To balance the indications of all three predictors, the position of each biological target on generated lists of potential biological targets was averaged; then, the obtained result was normalized so that it was represented by a number in the range from 0 (lowest probability) to 1 (most likely activity). Scores collected for selected compounds are presented in Table 1 (prediction results for all compounds in Supplementary Materials, Table S2).

To complement the predictions, we performed molecular docking for all tested compounds to H_3_R (homology model [59,60]), D_2_R (PDB: 7DFP), AChE (PDB: 6O4W), and BChE (PDB: 4BDS). Using two molecular docking programs, Glide 7.5 [61] and GOLD 5.3 [62], the binding of the tested derivatives to the selected proteins was assessed using the consensus score calculated as the average value of the normalized scores of individual scoring functions. The values of the collected consensus scores are presented in Table 1.

Molecular docking results were characterized by a much smaller scatter of values compared to those acquired from biological target predictors. A comparison of the obtained binding modes for different biological targets revealed intriguing similarities in the key binding elements of the ligands.

**Figure 3:**
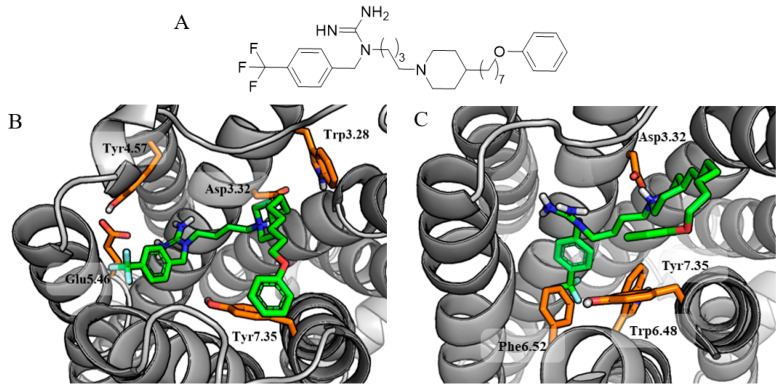
Structure (**A**) and predicted conformations of compound **25**, which was highly rated by target predictors and molecular docking, within binding sites of H_3_R (**B**) and D_2_R (**C**).

**Figure 4:**
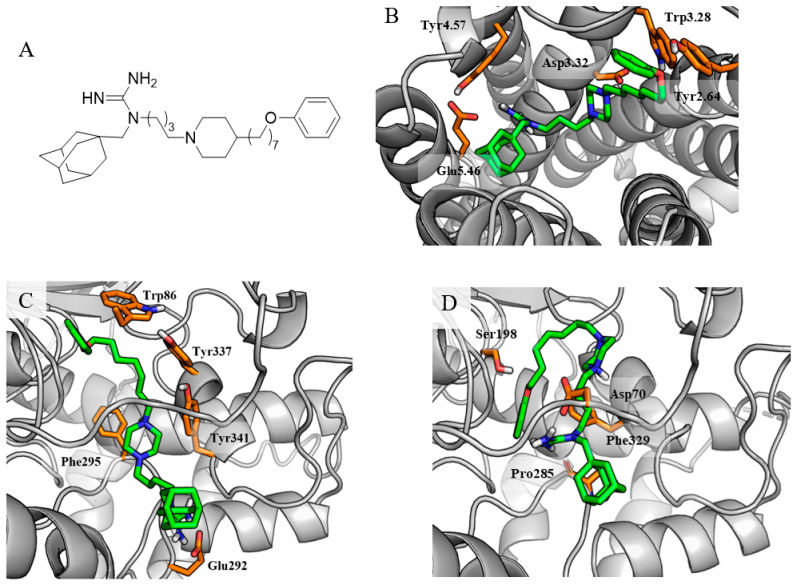
Structure of the strongest BChE inhibitor, compound **16** (**A**), and its binding mode to H_3_R (**B**), AChE (**C**), and BChE (**D**).

The common pattern of interactions is particularly evident in the binding modes obtained during docking to the H_3_R and D_2_R. The same placement of the *N*-benzyl-*N’*-[ω-(piperazin-1-yl)alkyl]guanidine fragment in the binding sites of both receptors shows that it can be a good leading element for the further design of multitarget H_3_R/D_2_R ligands. In the case of the H_3_R, this fragment is responsible for the formation of salt bridges with the key amino acids Asp3.32 and Glu5.46, as well as cation–π interactions with the aromatic rings of Tyr4.57 and Trp3.28. Moreover, the phenoxy group of the ligand creates additional interactions within the extracellular allosteric site, i.e., aromatic interactions with Tyr7.35 observed for compound **25** (Figure 3B) or hydrogen bond with Tyr2.64 in the case of compound **16** (Figure 4B). The very similar conformation of the main fragment of the compounds bound to the D_2_R ensures an analogous interaction with Asp3.32 and aromatic interactions with Trp6.48, Phe6.52, and Tyr7.35 (Figure 3C).

When docking to cholinesterases, the differences resulting from the size of the active sites of both enzymes are clearly visible. The most common binding mode of the tested compounds to AChE was the arrangement in which the *N*-[ω-(piperazin-1-yl)alkyl]guanidine fragment was located at the entrance to the enzyme gorge, participating in a salt bridge (guanidine—Glu292) and cation-π interactions with Tyr341, Tyr337, and Phe295 (Figure 4C). The long hydrophobic phenoxyalkyl substituent was extended along the enzyme active site, creating aromatic interactions with Trp86. Despite the promising values of the scoring function, such a binding mode would indicate significant exposure of the hydrophobic substituents at the guanidine core to the solvent surrounding the enzyme, which may explain the later experimental results.

The active site of BChE is much larger than that of the AChE, allowing almost the entire molecule to fit inside it. In compound **16**, the *N*-[1-adamantylmethyl]-*N*′-[4-(piperazin-1-yl)butyl]guanidine fragment creates an ionic bond (charged nitrogen of piperazine) and a salt bridge (guanidine moiety) with Asp70 and thus blocks the entrance to the active site (Figure 4D). Guanidine is additionally engaged in hydrogen bonds with Pro285 in the peripheral anionic site. The hydrophobic fragment of the ligand is located deeper within the enzyme cavity, creating aromatic interactions with Phe329, additionally stabilized by the H-bond with Ser198.

Considering the tested compounds as potential therapeutics in the fight against ASD, the ability to penetrate the blood-brain barrier (BBB) is an essential feature. The inherent physicochemical properties of guanidine-containing compounds often pose challenges for their penetration through the BBB. Despite this, guanidines play a vital role in the development of drugs that target the central nervous system. Numerous guanidine-based ligands have been determined to effectively penetrate the BBB despite the potential limitations [63,64,65]. To assess *N*-[ω-(piperazin-1-yl)alkyl]guanidine derivatives as thoroughly as possible at an early stage of the research, we used the SwissADME [66] service to determine their physicochemical properties (http://www.swissadme.ch/, accessed on 29 October 2024). As shown in Table 2, the most concerning properties are the relatively high LogP values and the large number of rotatable bonds, which in some cases led to negative indications of the BBB permeability predictor. Table S3 (Supplementary Materials) contains a complete set of physicochemical properties calculated using SwissADME for all compounds.

Compounds **17**, **22**, and **24** (predicted by SwissADME as CNS+, CNS+, and CNS−, respectively) have previously shown in vivo activity, suggesting their capability to cross the BBB and influence the central nervous system [53]. Following subcutaneous injection, there was a notable decrease in food consumption observed in rats. This behavior aligns with the known effects of blocking H_3_R in the central nervous system, and the decrease in consumption was similar to that observed with ciproxifan, a widely studied H_3_R antagonist/inverse agonist known to cross the blood-brain barrier. Although SwissADME predictions on the physicochemical and pharmacokinetic properties are useful for ligand optimizing, it is important to consider further factors, such as active transport mechanisms, which might influence in vivo activity.

Based on the results of the in silico screening, we selected 5 ligands with probable activity at the D_2_R and 11 which may be potential cholinesterase inhibitors for experimental evaluation.

At the screening concentrations of 1 µM and 100 nM, two compounds (**25** and **30**) exhibited a notable inhibition of [^3^H]methylspiperone binding at human D_2_R expressed on HEK-293 cells, with a reduction of over 50% at the 1 µM concentration. The remaining three compounds showed a moderate level of affinity, with inhibition percentages ranging from 31.8% to 45.6% at the same concentration. For the two most active compounds, radioligand displacement assays were also carried out on Chinese hamster ovary (CHO) cells expressing human D_2s_R or D_3_R. The experimental outcomes corroborated the comparable affinity of compounds **25** and **30** to the D_2_ dopamine receptor (p*K*_i_ = 6.22 and 6.12, respectively). Moreover, compound **30** revealed a greater selectivity for the D_2_ over the D_3_ receptor. Detailed information on the activity of the tested compounds towards the D_2_R is compiled in Table 3.

We also evaluated the inhibitory potency of the selected compounds against e*lectric eel* AChE (*ee*AChE) and *equine serum* BChE (*es*BChE) using Ellman’s test protocol at a screening concentration of 10 μM. For the most potent compounds that displayed at least 50% enzyme inhibition, we determined their IC_50_ values. Table 4 summarizes the activity of the compounds selected from the virtual screening against acetyl- and butyrylcholinesterase. The activity levels for all tested compounds against AChE were found to be low, while seven compounds showed a moderate ability to inhibit BChE.

## 3. Methods

### 3.1. Computer-Aided Evaluation of Ligands

To identify compounds with potential activity towards the desired biological targets—dopamine D_2_ receptor (D_2_R), acetylcholinesterase (AChE), and butyrylcholinesterase (BChE)—a two-step evaluation was performed. Both a ligand-based assessment with biological target predictors and an analysis of ligand interaction with biological targets were applied.

To predict the protein targets with the highest likelihood for each ligand, the assessment initially employed the SwissTargetPrediction (STP) (http://www.swisstargetprediction.ch/, accessed on 29 October 2024), SEA (https://sea.bkslab.org/, accessed on 29 October 2024), and PPB2 tools (https://ppb2.gdb.tools/, accessed on 29 October 2024). The SMILES code corresponding to the neutral forms of the ligands were used as an input. A ranked list of potential biological targets was generated by each predictor. In order to address the variability in the number of biological targets identified by different predictors and different scales, and to prioritize high-probability targets, only the initial 15 designated proteins (referred to as “top 15”) were taken into consideration. The position of the indicated activity towards the D_2_R, AChE, and BChE among the top 15 was recorded for each ligand. To achieve an equal participation of each predictor in the final assessment of potential activity, we computed the average position of a specific biological target on the ranking list for each ligand. Positions beyond the top 15 were assigned a consistent value of 16 in order to factor them into the average. In order to simplify the comparison of the averaged ranking with other results, min-max normalization was utilized according to Equation (1). This ensured that the averaged poses were assigned values ranging from 0 to 1. A value of 0 represented the biological target ranked the last among the top 15 in all predictors, while a value of 1 specified the biological target identified as the most likely by all predictors.

(1) $$y=\left(x-min_{rank}\right)/\left(max_{rank}\;-min_{rank\;}\right)$$

where x—average position of biological target on SwissTargetPrediction, SEA, and PPB2 ranking lists, $min_{rank}$—16, and $max_{rank}$—1.

The molecular docking of ligands to the binding sites of the dopamine D_2_R, AChE, and BChE constituted the second component of the assessment. The docking process involved the utilization of two distinct programs: Glide (Maestro, Schrödinger, LLC, New York, NY, USA) and GOLD (CCDC, Cambridge, UK). The LigPrep 4.2 tool was used to prepare all ligands, assigning them appropriate partial charges for pH 7.4 and generating potential stereoisomers and tautomers. The D_2_R (PDB: 7DFP), AChE (PDB: 6O4W), and BChE (PDB: 4BDS) complexes were obtained from the PDB database. Additionally, docking experiments were conducted with H_3_R to provide a more comprehensive characterization of the multitarget ligands. As there were no experimental structures accessible at the time of the computational experiments, a homology model that had been previously published was utilized [59,60]. Depending on the program, a different protocol was employed to prepare proteins for docking. The Hermes 1.7 tool was used to prepare each complex for docking with the GOLD program. The water molecules and ligands were extracted from each protein, hydrogen atoms were added, and histidine protonation at HE2 positions was confirmed. The docking sites were determined by considering all amino acids surrounding the co-crystallized ligand atoms within a given radius. The individual values for each protein were as follows: D_2_R—10 Å radius from spiperone (SIP), AChE—10 Å radius from donepezil (DOP), and BChE—12 Å radius from tacrine (THA) and 22 Å from the carbon alpha (CA) atom of Asp3.32 from H_3_R homology model. Docking to D_2_R and H_3_R involved the use of the GoldScore evaluation function, whereas ChemScore was employed for acetylcholinesterase and butyrylcholinesterase. Ten docking results were obtained for each ligand. When docking with the Glide program, proteins were prepared using the Protein Preparation Wizard 5.7 tool. This involved adding any missing hydrogen atoms, rebuilding disulfide bridges, removing water molecules, and assigning appropriate charges for a pH of 7.4. A grid was created for each protein, with the center positioned at the centroid of the ligand or, in the case of the histamine H_3_R, at the CA site, specifically the Asp3.32 atom. All grids were sized to dock ligands of 25 Å or smaller. During docking, the XP protocol was used and the 10 best poses were collected for each ligand. Following the assessment, all findings were gathered and standardized using the min-max technique for each individual biological target according to Equation (2). Table 5 summarizes the minimum and maximum docking scores achieved for each target and both docking programs. The consensus score was calculated by averaging the normalized values of the best ligand poses obtained in both docking runs.

(2) $$y=\left(x-min_{ds}\right)/\left(max_{ds}-min_{ds\;}\right)$$

where x—docking score of ligand top scored pose, $min_{ds}$—the lowest docking score among all generated poses, and $max_{ds}$—the highest docking score among all generated poses.

The predictor score values (normalized means) were used as an initial indicator of whether the compounds could interact with D_2_R, AChE, or BChE. Further, the preselection of ligands was based on docking simulations and the following cutoffs for means of normalized scoring function values: D_2_R—mean D_2_R docking score ≥ 0.6 and cholinesterases—mean AChE docking score ≥ 0.48. As we could test more compounds against cholinesterases than toward D_2_R, we finally chose 5 compounds for the D_2_R assay and 11 compounds for Ellman’s assay, based on the established cutoffs, consistency of binding poses, and immediate availability for testing.

### 3.2. Inhibition of Electric eel AChE and Equine Serum BChE

To assess the cholinesterase inhibitory activities of the target compounds, a modified Ellman’s method [69] was employed, which had been adapted for 96-well microplates as previously described [70]. The percentage of enzyme inhibition was determined using a screening concentration of 10 μM. Compounds demonstrating a minimum of 50% inhibition underwent additional testing to determine IC_50_ values, utilizing absorbance data derived from six different inhibitor concentrations. All experiments were performed in triplicate and tacrine was used as a reference compound.

### 3.3. In Vitro Screening Against Human D_2_ Receptor

The initial in vitro screening with estimation of % inhibition of the binding of [^3^H]methyl-spiperone for D_2_R was determined as described previously [71]. The experiments were commercially performed by Eurofins Cerep (Celle-Lévescault, France), using their own cell lines. Human recombinant dopamine D_2_R expressed in HEK-293 cells was used in modified Tris-HCl buffer pH 7.4. A 20 μg aliquot was incubated with 0.3 nM [^3^H]methyl-spiperone for 60 min at 25 °C. Non-specific binding was estimated in the presence of 10 μM butaclamol. Receptor proteins were filtered and washed; the filters were then counted to determine whether [^3^H]methyl-spiperone specifically bound. Five compounds were tested at 1 µM and 100 nM concentrations. Compound binding was calculated as a % inhibition of the binding of a [^3^H]methyl-spiperone.

### 3.4. In Vitro Human D_2_ and D_3_ Receptor Radioligand Displacement Assay

Radioligand binding studies of the new compounds **25** and **30** were performed on membrane fractions of CHO-K1 cells stably expressing human D_2short_R or D_3_R, as described previously [72]. The cell lines came from the laboratory of Prof. Stark. [^3^H]spiperone (0.2 nM) served as the radioligand, non-specific binding was determined with 10 μM haloperidol, and inhibition constant (*K*_i_) values were derived using the Cheng–Prusoff equation (Equation (3)):

(3) $$K_{i}=IC_{50}/\left(1+\frac{L}{K_{d}}\right)$$

where L—concentration of [^3^H]spiperone, $IC_{50}$—values determined by nonlinear regression, and $K_{d}$—dissociation constant

## 4. Conclusions

**Figure 5:**
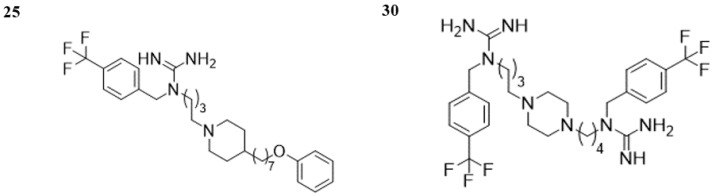
Structures of novel multitarget-directed ligands **25** and **30**, combining activity against H_3_R and D_2_R.

In summary, we report the identification of novel multitarget-directed ligands from the group of (ω-piperazin-1-ylalkyl)guanidine derivatives through virtual screening. Compounds **25** and **30** (Figure 5), which have demonstrated significant activity towards H_3_R (pA_2_ = 7.90 and 7.99, respectively [53]) in previous studies, also exhibited notable binding ability to the D_2_R, displacing over 50% of the radioligand at a screening concentration of 1 µM. Additional tests revealed more selective binding of compound **30** to D_2_R (p*K*_i_ = 6.12) over D_3_R (p*K*_i_ = 4.84) receptors than that of compound **25**. These compounds might serve as a promising starting point for the rational development of new multifunctional ligands, offering potential for the introduction of a new and effective therapy for ASD.

## Figures and Tables

**Table 1:** Affinities of compounds at H_3_R and predicted probability of affinities at D_2_R and cholinesterases based on normalized scores obtained from biological target predictors and molecular docking results.

	pA2 *	Target Predictors ^a^			Molecular Docking ^b^		
Cmp.	H3R	D2R	AChE	BChE	D2R	AChE	BChE
**6**	7.84	0.60	0.13	0.00	0.47	0.48	0.67
**7**	7.39	0.53	0.07	0.00	0.50	0.50	0.73
**8**	7.59	0.60	0.07	0.00	0.53	0.55	0.68
**16**	7.28	0.27	0.13	0.07	0.44	0.73	0.44
**17**	8.21	0.53	0.13	0.05	0.53	0.73	0.51
**18**	7.98	0.40	0.07	0.07	0.67	0.72	0.73
**22**	7.80	0.60	0.00	0.00	0.69	0.70	0.85
**25**	7.90	0.80	0.40	0.27	0.70	0.75	0.55
**27**	7.30	0.20	0.00	0.00	0.58	0.74	0.59
**28**	7.97	0.53	0.00	0.00	0.60	0.78	0.65
**29**	8.10	0.47	0.13	0.07	0.65	0.73	0.55
**30**	7.99	0.27	0.00	0.00	0.88	0.39	0.64
**31**	5.78	0.53	0.00	0.00	0.48	0.79	0.56

^a^ normalized mean position from three independent target predictors; ^b^ mean value of normalized docking score from two independent molecular docking procedures; * the negative logarithm of the molar concentration of the tested antagonist, which causes a twofold shift in the concentration–response curve for (R)-α-methylhistamine on electrically contracting guinea pig jejunum [52].

**Table 2:** Physicochemical properties and BBB permeability (passive diffusion), predicted by the SwissADME service.

Cmp.	MW	RB	TPSA	LogP	BBB
**6**	375.6	14	77.61	2.59	No
**7**	389.6	15	77.61	2.95	No
**8**	403.6	16	77.61	3.31	Yes
**16**	537.8	17	68.82	5.35	No
**17**	479.7	17	68.82	4.37	Yes
**18**	493.7	17	68.82	4.65	Yes
**22**	514.2	17	68.82	4.89	Yes
**25**	546.7	18	65.58	6.61	No
**27**	504.7	17	81.12	4.43	No
**28**	547.7	19	63.62	5.60	No
**29**	479.7	18	63.62	4.55	Yes
**30**	628.7	18	112.70	4.62	No
**31**	466.7	18	24.94	6.17	No

MW—molecular weight [g/mol]; RB—num. of rotatable bonds; TPSA—topological polar surface area [Å^2^]; LogP—consensus LogP_o/w_ for 5 calculation methods: iLOGP, XLOGP3, WLOGP, MLOGP, SILICOS-IT; BBB—blood–brain barrier permeability according to the BOILED-Egg model [67].

**Table 3:** Results of radioligand binding assay at human dopamine D_2_ and D_3_ receptor subtypes.

	D_2_R %inh. (1 µM) ^a^	D_2_R %inh. (100 nM) ^a^	D_2s_R p*K*_i_ ± SEM ^b^	D_3_R p*K*_i_ ± SEM ^b^
**18**	45.6	10.1	nd	nd
**22**	32.0	−1.0	nd	nd
**25**	53.2	11.7	6.22 ± 0.07	5.91 ± 0.03
**29**	31.8	6.3	nd	nd
**30**	54.5	17.2	6.12 ± 0.09	4.84 ± 0.06
**AY23028**	nd	nd	9.14 ^a^	8.64 ^c^

^a^ Human D_2_ receptor binding assay with antagonist radioligand [^3^H]methylspiperone using HEK293 cell line expressed as percent specific binding inhibition of control; ^b^ displacement assay using membrane suspension of CHO cell line expressing human D_2s_ and CHO cell line expressing human D_3_ with [^3^H]spiperone; nd, not determined. ^c^ Literature data [68].

**Table 4:** Inhibition of *ee*AChE and *es*BChE by selected compounds.

	*ee*AChE %inh. (10 µM) ^a^	*ee*AChE IC_50_ [µM] ^b^ (pIC_50_)	*es*BChE %inh. (10 µM) ^a^	*es*BChE IC_50_ [µM] ^c^ (pIC_50_)
**6**	11.2 ± 5.1	nd	28.4 ± 1.4	nd
**7**	13.2 ± 4.7	nd	29.0 ± 3.7	nd
**8**	13.5 ± 5.9	nd	35.4 ± 4.2	nd
**16**	22.1 ± 2.9	nd	79.9 ± 1.8	3.47 ± 0.11 (5.46)
**17**	17.9 ± 3.4	nd	66.0 ± 1.4	6.37 ± 0.27 (5.20)
**18**	18.2 ± 5.3	nd	79.1 ± 0.9	3.71 ± 0.11 (5.43)
**22**	26.9 ± 2.2	nd	80.6 ± 1.2	3.53 ± 0.13 (5.45)
**27**	13.8 ± 6.9	nd	71.5 ± 1.8	4.75 ± 0.16 (5.32)
**28**	16.4 ± 8.1	nd	76.7 ± 1.3	3.87 ± 0.13 (5.41)
**29**	23.3 ± 3.5	nd	76.3 ± 2.0	4.00 ± 0.14 (5.40)
**31**	6.3 ± 2.3	nd	20.6 ± 0.5	nd
**Tacrine**	-	0.024 ± 0.001 (7.62)	-	0.015 ± 0.001 (7.82)

^a^ mean value ± standard deviation (SD) of three independent experiments; ^b^ IC_50_ mean value ± standard error of the mean (SEM) of triplicate independent experiments on *electric eel* AChE; ^c^ IC_50_ mean value ± standard error of the mean (SEM) of triplicate independent experiments on BChE from *equine serum*; nd, not determined.

**Table 5:** Min and max docking scores for each target and program.

	GOLD $(min_{ds}$$/max_{ds}$)	Glide * $(min_{ds}$$/max_{ds}$)
D_2_R	64.98/91.72	−4.14/−11.14
AChE	35.63/52.45	−2.46/−11.74
BChE	67.71/100.89	−3.36/−11.33

* Due to the inverse scale with negative values used in Glide score, absolute values were used for calculations.

## Data Availability

Data are contained within the article and Supplementary Materials.

## Supplementary Materials

The supporting information can be downloaded at https://www.mdpi.com/article/10.3390/molecules29225271/s1. The supporting information contains compound structures (Table S1); detailed activity prediction results (Table S2); and physicochemical property prediction results (Table S3).

## Abbreviations

AChE: acetylcholinesterase; ADHD, Attention Deficit Hyperactivity Disorder; ASD, autism spectrum disorder; BTBR, Black and Tan Brachyury; BBB, blood–brain barrier; BChE, butyrylcholinesterase; CDS, Centers for Disease Control; CNS, central nervous system; CHO, Chinese hamster ovary; D_2_R, dopamine D_2_ receptor; *ee*AChE, *electric eel* AChE; *es*BChE *equine serum* BChE; GABA, gamma-aminobutyric acid; H_3_R, histamine H_3_ receptor; HEK, Human Embryonic Kidney; Ki, inhibition constant; MBB, marble-burying behavior; MTDLs, multitarget-directed ligands; mRNA, messenger ribonucleic acid; SNPs, single-nucleotide polymorphisms.
