# Supplementary material for: Virtual Screening Approaches to Identify Promising Multitarget-Directed Ligands for the Treatment of Autism Spectrum Disorder
Source: Molecules. 2024 Nov 7;29(22):5271. doi: 10.3390/molecules29225271 (PMC11596355; doi:10.3390/molecules29225271)
Supplement: Supplementary file 1 [file molecules-29-05271-s001.zip › molecules-3261256-supplementary.pdf]

Supplementary Materials

Virtual Screening Approaches to Identify Promising Multitarget-Directed Ligands for the Treatment of Autism Spectrum Disorder

Jakub Jończyk [1,2], Klaudia Przybylska [1], Marek Staszewski [3], Justyna Godyń [1], Tobias Werner [4], Monika Stefaniak-Napieralska [3], Holger Stark [4], Krzysztof Walczyński [3] and Marek Bajda [1,*]

1 Department of Physicochemical Drug Analysis, Faculty of Pharmacy, Jagiellonian University Medical College, 30-688 Kraków, Poland
2 Sano—Centre for Computational Medicine, 30-054 Kraków, Poland
3 Department of Synthesis and Technology of Drugs, Faculty of Pharmacy, Medical University of Lodz, 90-151 Lodz, Poland
4 Institute of Pharmaceutical and Medicinal Chemistry, Heinrich Heine University, 40225 Düsseldorf, Germany
* Correspondence: marek.bajda@uj.edu.pl

Supporting Information:

- **Compound structures (Table S1)**
- **Detailed activity prediction results (Table S2)**
- **Physicochemical properties prediction results (Table S3)**

Table S1. Chemical structures of tested ligands.

General structure for compounds 1-24

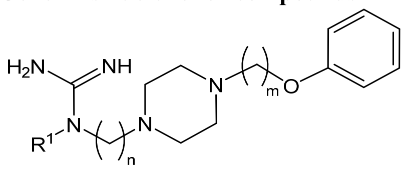

Cmp.	R^1	n	m
**1**	H	3	2
**2**	H	3	3
**3**	H	3	4
**4**	H	3	5
**5**	H	3	6
**6**	H	3	7
**7**	H	3	8
**8**	H	3	9
**9**	H	2	7
**10**	H	4	7
**11**	H	5	7
**12**	H	6	7
**13**	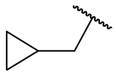	4	7
**14**	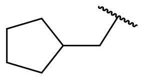	4	7
**15**	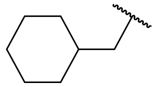	4	7

**16**	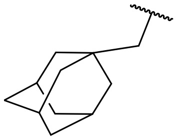	4	7
**17**	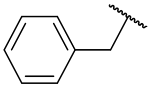	4	7
**18**	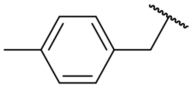	4	7
**19**	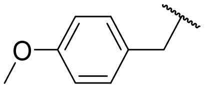	4	7
**20**	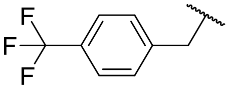	4	7
**21**	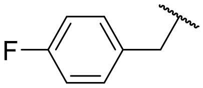	4	7
**22**	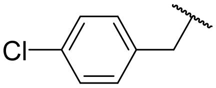	4	7
**23**	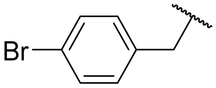	4	7
**24**	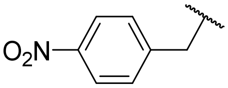	4	7

**Cmp.**	**Structure**	**n**	**m**
**25**	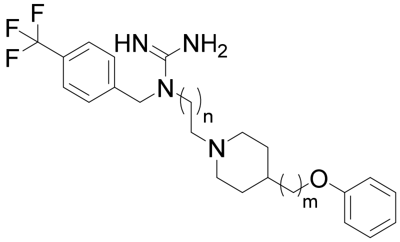	3	7
**26**	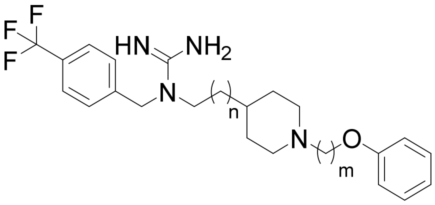	3	7
**27**	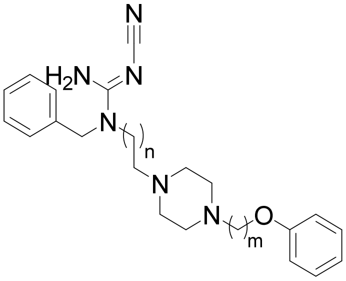	3	7

**28**	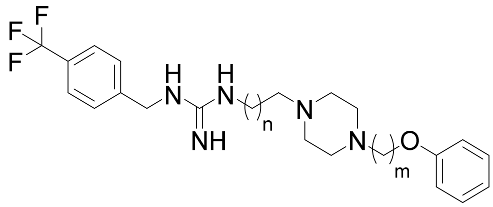	3	7
**29**	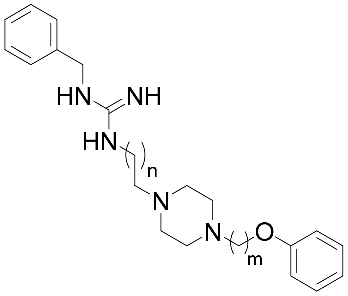	3	7
**30**	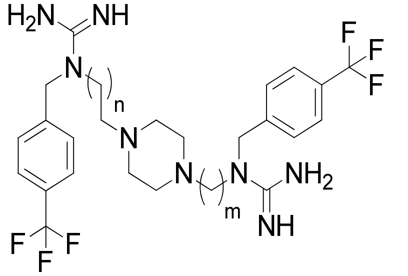	3	4
**31**	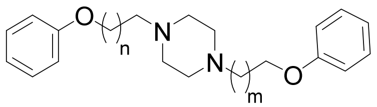	6	6
**32**	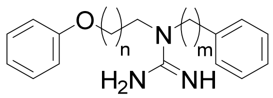	6	1

Table S2. Affinities of tested compounds against histamine H_3 receptor and predicted probability of affinities at dopamine D_2 receptor and cholinesterases based on normalized scores (NS) obtained from biological target predictors and molecular docking results.

	pA_2[*]	Target predictors												Molecular docking								
	H_3R	D_2R				AChE				BChE				D_2R			AChE			BChE		
		STP	SEA	PPB2	NS[a]	STP	SEA	PPB2	NS[a]	STP	SEA	PPB2	NS[a]	Glide	GOLD	NS[b]	Glide	GOLD	NS[b]	Glide	GOLD	NS[b]
**1**	6.61	16	4	7	0.47	16	16	15	0.00	16	16	16	0.00	-6.35	73.21	0.31	-9.26	37.83	0.43	-7.61	83.36	0.50
**2**	6.85	16	4	4	0.53	16	16	15	0.00	16	16	16	0.00	-6.36	73.37	0.32	-7.97	36.74	0.33	-8.07	81.54	0.50
**3**	7.26	16	2	5	0.53	16	16	13	0.07	16	16	15	0.00	-6.92	78.02	0.44	-9.07	38.53	0.44	-8.32	80.91	0.51
**4**	7.22	16	2	3	0.60	16	16	13	0.07	16	16	16	0.00	-7.04	80.84	0.50	-9.04	39.24	0.46	-8.54	82.52	0.55
**5**	7.68	16	2	4	0.60	16	16	11	0.13	16	16	16	0.00	-7.11	82.43	0.54	-7.84	40.85	0.44	-9.123	86.68	0.65
**6**	7.84	16	2	4	0.60	16	16	11	0.13	16	16	16	0.00	-7.30	77.99	0.47	-8.40	40.92	0.48	-8.625	90.01	0.67
**7**	7.39	16	1	6	0.53	16	16	13	0.07	16	16	16	0.00	-7.19	80.19	0.50	-8.62	41.39	0.50	-9.16	91.88	0.73
**8**	7.59	16	2	4	0.60	16	16	13	0.07	16	16	16	0.00	-7.87	78.84	0.53	-8.36	43.37	0.55	-8.31	92.19	0.68
**9**	7.69	16	1	3	0.60	16	16	12	0.07	16	16	16	0.00	-7.54	73.65	0.41	-7.44	39.66	0.39	-8.99	80.39	0.54
**10**	8.00	16	2	3	0.60	16	16	12	0.07	16	16	15	0.00	-7.62	83.17	0.59	-8.28	40.16	0.45	-9.13	78.17	0.52
**11**	7.96	16	2	3	0.60	16	16	10	0.13	16	16	16	0.00	-7.64	78.54	0.50	-7.90	40.23	0.43	-8.89	87.41	0.64
**12**	7.67	16	2	4	0.53	16	16	13	0.07	16	16	14	0.07	-7.14	84.53	0.58	-8.34	41.93	0.50	-7.99	83.15	0.52
**13**	8.06	9	14	4	0.47	16	16	14	0.07	16	16	14	0.07	-8.16	78.02	0.53	-8.95	46.27	0.67	-9.21	78.88	0.54
**14**	8.08	16	13	9	0.20	16	16	15	0.00	16	16	13	0.07	-8.04	80.75	0.57	-8.66	47.96	0.70	-9.09	100.89	0.86
**15**	8.04	16	13	7	0.27	16	16	14	0.07	16	16	15	0.00	-9.22	81.75	0.68	-7.54	48.07	0.64	-9.52	73.79	0.48
**16**	7.28	16	16	5	0.27	16	16	13	0.13	16	16	13	0.07	-7.11	76.99	0.44	-7.62	50.82	0.73	-3.5	96.56	0.44
**17**	8.21	8	13	4	0.53	16	12	13	0.13	16	15	13	0.07	-7.77	79.26	0.53	-8.85	48.51	0.73	-8.54	80.29	0.51
**18**	7.98	16	13	2	0.40	16	12	16	0.07	16	14	15	0.07	-7.49	88.15	0.67	-8.34	49.21	0.72	-10.17	87.7	0.73
**19**	7.69	16	6	6	0.47	16	14	15	0.07	16	16	15	0.00	-8.14	76.82	0.51	-8.09	46.21	0.62	-7.12	89.31	0.56
**20**	8.49	4	16	3	0.53	11	16	16	0.13	16	16	16	0.00	-9.65	79.58	0.67	-10.37	42.24	0.62	-11.32	94.29	0.90
**21**	7.89	8	6	3	0.67	16	16	16	0.00	16	16	15	0.00	-8.36	91.72	0.80	-9.91	44.75	0.67	-8.47	81.84	0.53
**22**	7.80	8	9	3	0.60	16	16	16	0.00	16	16	16	0.00	-8.29	85.86	0.69	-8.45	48.16	0.70	-9.25	99.49	0.85
**23**	7.80	16	16	4	0.27	16	13	16	0.07	16	15	15	0.07	-9.99	87.14	0.83	-10.07	48.7	0.80	-10.37	84.94	0.70
**24**	8.43	16	16	2	0.33	16	16	12	0.07	16	16	16	0.00	-9.09	82.33	0.68	-9.2	45.68	0.66	-10.09	88.12	0.73
**25**	7.90	3	7	3	0.80	8	9	13	0.40	9	16	12	0.27	-8.78	84.54	0.70	-10.01	47.29	0.75	-8.55	82.94	0.55

**26**	8.35	11	7	6	0.53	7	9	13	0.40	9	16	13	0.20	-9.82	82.44	0.73	-10.51	44.66	0.70	-11.33	98.62	0.97
**27**	7.30	16	16	8	0.20	16	16	16	0.00	16	16	15	0.00	-8.44	79.53	0.58	-9.01	48.7	0.74	-8.11	87	0.59
**28**	7.97	16	3	5	0.53	16	16	15	0.00	16	16	16	0.00	-9.11	78.3	0.60	-11.74	44.91	0.78	-8.70	88.3	0.65
**29**	8.10	16	3	7	0.47	16	16	10	0.13	16	16	12	0.07	-8.14	84.36	0.65	-9.94	46.6	0.73	-7.77	85.79	0.55
**30**	7.99	16	16	6	0.27	16	16	16	0.00	16	16	16	0.00	-11.14	85.47	0.88	-9.10	36.79	0.39	-8.15	90.14	0.64
**31**	5.78	16	6	3	0.53	16	16	15	0.00	16	16	16	0.00	-7.69	77.21	0.48	-7.85	52.45	0.79	-6.98	89.57	0.56
**32**	5.78	7	16	16	0.27	10	16	16	0.13	9	16	16	0.13	-6.33	69.08	0.23	-6.98	43.01	0.46	-6.81	87.82	0.52

[a] Normalized mean position from three independent target predictors.

[b] Mean value of normalized docking score from two independent molecular docking procedures.

[*] The negative logarithm of the molar concentration of the tested antagonist, which causes a twofold shift of the concentration-response curve for (R)-α-methylhistamine on electrically contracting guinea pig jejunum.

Table S3. Physicochemical parameters and BBB permeability prediction calculated using the SwissADME service.

	MW	RB	TPSA	LogP	BBB
**1**	305.42	9	77.61	0.95	No
**2**	319.45	10	77.61	1.37	No
**3**	333.47	11	77.61	1.58	No
**4**	347.50	12	77.61	1.95	No
**5**	361.52	13	77.61	2.30	No
**6**	375.55	14	77.61	2.59	No
**7**	389.58	15	77.61	2.95	No
**8**	403.60	16	77.61	3.31	Yes
**9**	361.52	13	77.61	2.33	No
**10**	389.58	15	77.61	2.90	No
**11**	403.60	16	77.61	3.28	Yes
**12**	417.63	17	77.61	3.55	Yes
**13**	443.67	17	68.82	3.85	Yes
**14**	471.72	17	68.82	4.48	Yes
**15**	485.75	17	68.82	4.79	Yes
**16**	537.82	17	68.82	5.35	No
**17**	479.70	17	68.82	4.37	Yes
**18**	493.73	17	68.82	4.65	Yes
**19**	509.73	18	78.05	4.35	No
**20**	547.70	18	68.82	5.46	No
**21**	497.69	17	68.82	4.64	Yes
**22**	514.15	17	68.82	4.89	Yes
**23**	558.60	17	68.82	5.00	Yes
**24**	524.70	18	114.64	3.69	No
**25**	546.71	18	65.58	6.61	No
**26**	546.71	18	65.58	6.54	No
**27**	504.71	17	81.12	4.43	No
**28**	547.70	19	63.62	5.60	No
**29**	479.70	18	63.62	4.55	Yes
**30**	628.70	18	112.70	4.62	No
**31**	466.70	18	24.94	6.17	No
**32**	339.47	12	62.34	3.97	Yes

MW - Molecular weight [g/mol]; RB - Num. rotatable bond; TPSA - Topological Polar Surface Area [Å^2]; LogP - Consensus $LogP_{o/w}$ for 5 calculation methods: iLOGP, XLOGP3, WLOGP, MLOGP, SILICOS-IT; BBB - blood–brain barrier permeability according BOILED-Egg model.
